# *HSPC117* Is Regulated by Epigenetic Modification and Is Involved in the Migration of JEG-3 Cells

**DOI:** 10.3390/ijms150610936

**Published:** 2014-06-17

**Authors:** Hong Ma, Mei-Yu Qi, Xu Zhang, Yue-Ling Zhang, Liang Wang, Zhong-Qiu Li, Bo Fu, Wen-Tao Wang, Di Liu

**Affiliations:** 1 Heilongjiang Academy of Agricultural Sciences Postdoctoral Program, Harbin 150086, Heilongjiang, China; E-Mail: joan7843@163.com; 2 Heilongjiang Academy of Agricultural Sciences, Harbin150086, Heilongjiang, China; E-Mails: zhangxu_19871119@163.com (X.Z.); zhang.yl@foxmail.com (Y.-L.Z.); wlwl448@163.com (L.W.); lizhongqiu1974@163.com (Z.-Q.L.); Fubohao810@sohu.com (B.F.); Wangwentao_1981@163.com (W.-T.W.)

**Keywords:** *HSPC117*, epigenetic modification, cell migration

## Abstract

The human hematopoietic stem/progenitor cell 117 (HSPC117) protein is an essential component of protein complexes and has been identified to be involved in many important functions. However, how this gene expression is regulated and whether the *HSPC117* gene affects cell migration is still unknown. The aim of this study was to identify whether *HSPC117* mRNA expression is regulated by epigenetic modification and whether *HSPC117* expression level affects the expression of matrix metalloproteinase 2 (*MMP 2*), matrix metalloproteinase 14 (*MMP 14*), and tissue inhibitor of metalloproteinases 2 (*TIMP 2*), and further affects human placenta choriocarcinoma cell (JEG-3) migration speed. In our epigenetic modification experiment, JEG-3 cells were cultured in medium with the DNA methyltransferase inhibitor 5-aza-2'-deoxycytidine (5-aza-dC), the histone deacetylase (HDAC) inhibitor trichostatin A (TSA), or both inhibitors. Then, the *HSPC117* mRNA and protein expressions were assessed using real-time quantitative PCR (qPCR) and Western blot assay. The results showed that, compared to the control, *HSPC117* mRNA expression was increased by TSA or 5-aza-dC. The highest *HSPC117* expression level was found after treatment with both 5-aza-dC and TSA. Further, in order to investigate the effect of *HSPC117* on *MMP 2*, *MMP 14*, and *TIMP 2* mRNA expressions, pEGFP-C1-HSPC117 plasmids were transfected into JEG-3 cells to improve the expression of *HSPC117* in the JEG-3 cells. Then, the mRNA expression levels of *MMP 2*, *MMP 14*, *TIMP 2*, and the speed of cell migration were assessed using the scratch wound assay. The results showed that over-expression of *HSPC117* mRNA reduced *MMP 2* and *MMP 14* mRNA expression, while *TIMP 2* mRNA expression was up-regulated. The scratch wound assay showed that the migration speed of JEG-3 cells was slower than the non-transfected group and the C1-transfected group. All of these results indicate that *HSPC117* mRNA expression is regulated by epigenetic modification; over-expression of *HSPC117* decreases *MMP 2* and *MMP 14* transcription, reduces cell migration speed, and increases *TIMP 2* transcription.

## 1. Introduction

The human hematopoietic stem/progenitor cell 117 (HSPC117) protein, also known as C22orf28 (with an analogous protein in Bacteria and Archaea, called RtcB), is a member of the UPF0027 family [1,2]. The HSPC117/RtcB protein has been determined to be an essential subunit of a tRNA ligase complex that is involved in tRNA splicing and other RNA repair reactions [3]. It has been demonstrated that this protein family has high sequence similarity with proteins in Eucarya, Bacteria, and Archaea [4]; for example, murine focal adhesion associated protein (FAAP), a homologous protein of HSPC117, is 99% identical to human HSPC117 [5]. Moreover, as an essential component of protein complexes, HSPC117 is also present in the TNF-α mRNA 3' AU-rich element binding complexes and osmotic response element binding protein KIAA0827 [6]. Previous studies have shown that FAAP interferes with the activation of mitogen activated protein kinase (MAPK) by inducing levels of extracellular signaling related kinase (ERK) dephosphorylation and/or reducing phosphorylation in mice [5]. Further, HSPC117 acts as an activator of Serum Response Factor (SRF), which is a transcription factor with important roles in the regulation of the actin cytoskeleton [7]. Recent studies showed that HSPC117 was important in embryo and placenta development [8]. Although HSPC117 has been identified to be involved in many important functions, research into this gene is still limited, and how this gene expression is regulated is still unknown.

Recent studies showed that *HSPC117* was expressed in mouse pre- and post-implantation embryos. When *HSPC117* RNAi knock-down embryos were transferred into pseudopregnant females, a large number of embryo deaths were observed after nine days of pregnancy [8]. This study showed that mouse *in vivo* produced (IVP) and somatic cell nuclear transfer (SCNT) blastocyst HSPC117 expression was significantly different. Further, placental abnormalities were found in HSPC117 RNAi and low expression embryos. Other studies showed that HSPC117 protein participates in the spreading initiation center (SIC) during the early stages of cell spreading [7] and in cell adhesion.

It is commonly stated that the efficiency of successful development of SCNT embryos is less than that of IVP embryos because of incomplete or error-prone epigenetic reprogramming [9]. Further, cell migration is involved in embryonic development and placental formation [10]. Thus, we speculate that *HSPC117* might be regulated by one or more epigenetic patterns and involved in cells migration.

Histone deacetylation and DNA methylation are important forms of epigenetic modification [11]. 5-aza-2'-deoxycytidine (5-aza-dC) can inhibit the activity of DNA methyl-transferase (DNMT), and trichostatin A (TSA) can inhibit non-competitively the activity of histone deacetylase (HDAC) [12]. In this study, we committed to characterize the regulation pattern of the *HSPC117* gene and analyze how TSA and 5-aza-dC influence the expression of the *HSPC117* gene. We have known that adherent cell movement is thought to be a result of a multi-factorial process, such as cell interactions with the extra-cellular matrix (ECM) and with adjacent cells [13]. The foundation of cell migration is the recognition and interaction between cells and specific extra-cellular matrix (ECM) components. Matrix metalloproteinase (MMPs) degrade ECM proteins, and create space for cell motility. Tissue inhibitor of metalloproteinases (TIMPs) effectively down-regulate the effect of MMPs. Both MMPs and TIMPs are involved in spatial and temporal ECM remodeling. HSPC117 is thought to regulate cell motility because it is specifically located in the early SIC at the time that cell adhesion occurs [14], and some experiments have proven that it affects cell adhesion through regulating vinculin-paxillin association [7]. However, there is a lack of data regarding HSPC117 alteration of the balance between MMPs and their inhibitors during the cell migration.

The scope of this paper was to characterize the regulation pattern of *HSPC117* and analyze the effect of TSA and 5-aza-dC on its expression level. Additionally, the expressions of *MMP 2*, *MMP 14*, and *TIMP 2*, and cell migration speed, when *HSPC117* was over-expressed, were observed to examine the association between *HSPC117* expression level and epigenetic modification, and cells migration.

## 2. Results and Discussion

### 2.1. Results

#### 2.1.1. Effect of Histone Deacetylation and Methylation on *HSPC117* Expression

**Figure 1:**
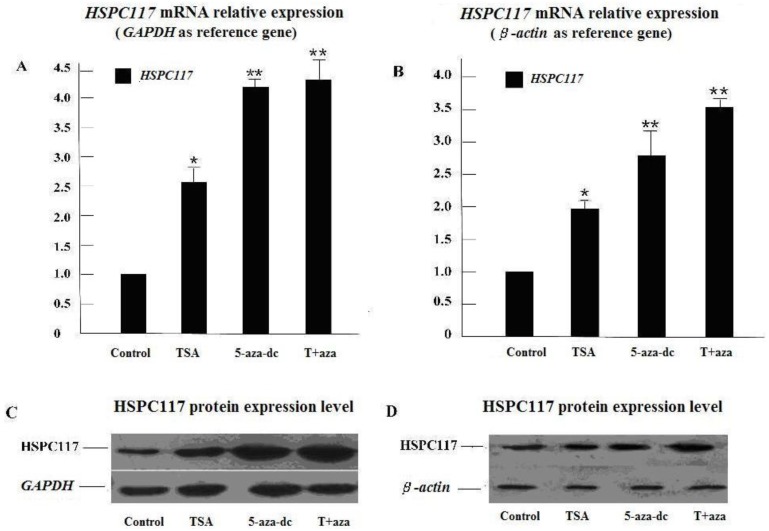
HSPC117 expressions in JEG-3 cells treated with TSA, 5-aza-dC or T + aza. (**A**) *HSPC117* mRNA expression (*GAPDH* as reference gene); (**B**) *HSPC117* mRNA expression (*β-actin* as reference). Each bar represents the mean of three independent experiments. (* *p* < 0.05, ** *p* < 0.01, compared to control, respectively); (**C**) HSPC117 protein expression detected by Western blot analysis. The GAPDH protein was used as a control; (**D**) HSPC117 protein expression deleted by Western blot analysis. The β-actin protein was used as a control.

To determine whether *HSPC117* mRNA and protein expressions are associated with epigenetic modification, we analyzed the relationship between TSA/5-aza-dC and *HSPC117* expression levels in human placental choriocarcinoma cell line (JEG-3) cells using qPCR and Western blot assays. As shown in Figure 1A, glyceraldehyde-3-phosphate dehydrogenase (*GAPDH*) as reference gene, the *HSPC117* mRNA expression level in each treatment group was higher than that of the control group. The expression level was about 4.2 times higher than that of the control group (*p* < 0.01) when cells were treated with a combination of both inhibitors (T + aza); this combination treatment group had the highest *HSPC117* transcriptional level. When cells were treated with 5-aza-dC or TSA separately, the *HSPC117* mRNA levels were about 4.0 times (*p* < 0.01) and 2.6 times (*p* < 0.05) higher than that of the control group, respectively. Similar results were obtained when *β-actin* was uesd as reference gene. When cells were treated with a combination of both inhibitors (T + aza) or 5-aza-dC or TSA separately, *HSPC117* mRNA levels were about 3.5, 2.7, and 1.9 times higher than that of the control group (Figure 1B). Further, the results of HSPC117 protein levels were similar (Figure 1C,D). The results suggest that *HSPC117* mRNA and protein expression could be epigenetically regulated by TSA, 5-aza-dC, or both.

#### 2.1.2. Differential Regulation of *HSPC117* on *MMP 2*, *MMP 14*, and *TIMP 2* Expressions in JEG-3 Cells Line

**Figure 2:**
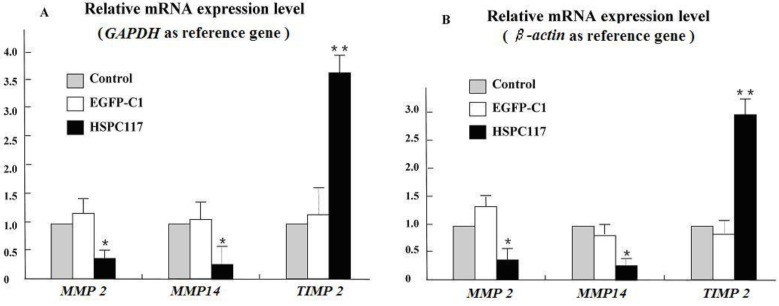
Relative mRNA expression of *MMP 2*, *MMP 14*, and *TIMP 2* in JEG-3 cell lines with over-expressed *HSPC117*. (**A**) *GAPDH* as reference gene; (**B**) *β-actin* as reference gene. (* *p* < 0.05, ** *p* < 0.01, compared to C1-transfected and control groups).

To determine whether *HSPC117* mRNA expression affects extra-cellular matrix (ECM), we detected *MMP 2*, *MMP 14*, and *TIMP 2* mRNA expressions in the JEG-3 cell lines with over-expressed *HSPC117* and C1-transfected cells *in vitro*. Basal expressions of *MMP 2*, *MMP 14*, and *TIMP 2* were also detected in the non-transfected JEG-3 cell lines, which were used as normal controls. As shown in Figure 2A (*GAPDH* as reference gene), qPCR analysis showed that *MMP 2* and *MMP 14* mRNA expression was slightly higher but that *TIMP 2* mRNA expression levels were slightly lower (*p* > 0.05) in the C1-transfected JEG-3 cell lines, compared to the control group; however, C1 plasmids had no significant effect on the expression of *MMP 2*, *MMP 14*, and *TIMP 2* mRNA. Expressions of *MMP 2* and *MMP 14* mRNA were significantly down-regulated in the JEG-3 cell lines with over-expressed *HSPC117*, which were only 0.46-fold (*p* < 0.05) and 0.31-fold (*p* < 0.05) of those in the normal JEG-3 cell lines, respectively. However, *TIMP 2* mRNA was up-regulated, which was 3.3-fold (*p* < 0.01) higher than that of the non-transfected group. Similar results were obtained when â-actin was used as reference gene. As shown in Figure 2B, *TIMP 2*, *MMP 2*, and *MMP 14* mRNA levels were about 2.8-, 0.42-, and 0.39-fold lower than that of the control group. These results showed that *MMP 2*, *MMP 14*, and *TIMP 2* mRNA expressions were significantly affected by the mRNA expression level of *HSPC117*. It is important to note that over-expression of the *HSPC117* gene changed the proportion of *MMP 2*, *MMP 14*, and *TIMP 2*. This meant a decrease in the amount of the TIMP 2/MMP 14/por-MMP 2 compound, as a clear result.

#### 2.1.3. Effect of *HSPC117* on JEG-3 Cell Migration and Wound Closure *in Vitro*

**Figure 3:**
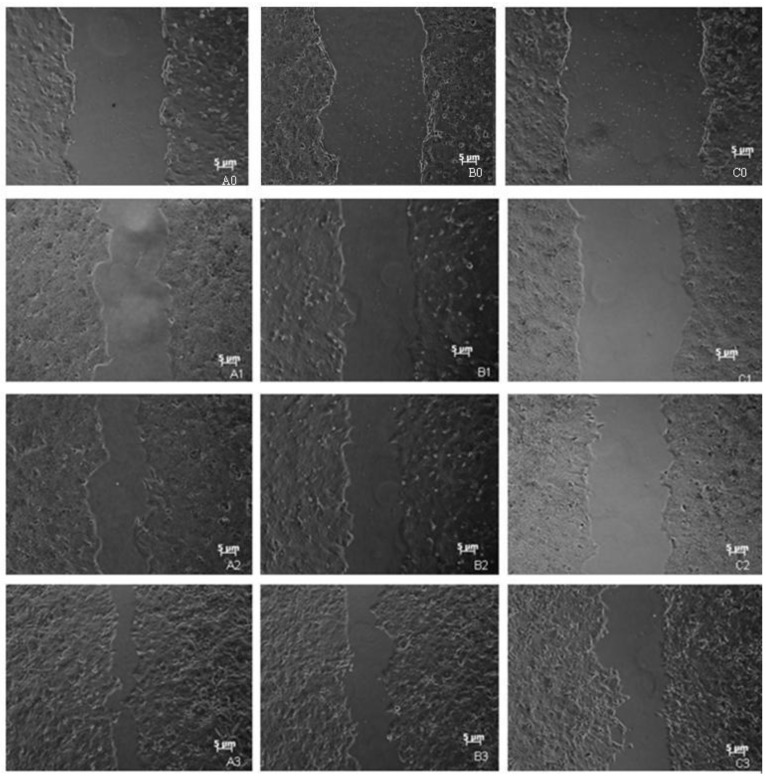
Wound distance analysis via scratch wound assays. **A0**, **A1**, **A2**, **A3**: images of control cells incubated for 0, 24, 48, and 72 h (50×); **B0**, **B1**, **B2**, **B3**: images of EGFP-C1 recombinant plasmid transfected cells incubated for 0, 24, 48 and 72 h (50×); **C0**, **C1**, **C2**, **C3**: images of EGFP-C1-HSPC117 recombinant plasmid transfected cells incubated for 0, 24, 48, and 72 h (50×).

**Figure 4:**
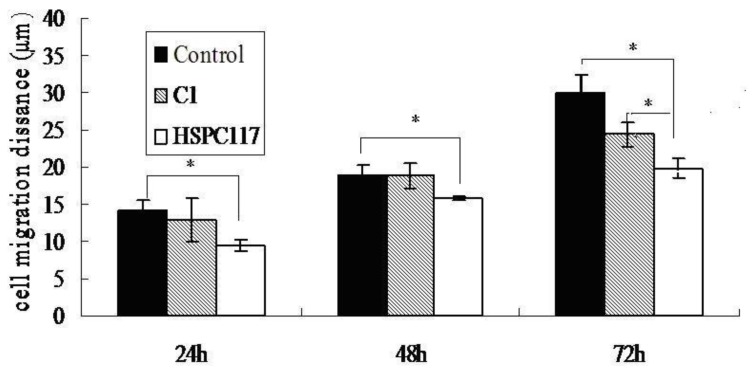
Cell migration distances of JEG-3 cells transfected with C1 or C1/HSPC117 plasmids at 24, 48, and 72 h (* *p* < 0.05, compared to non-transfected cells).

A significant attenuation of cell migration was observed in *HSPC117* over-expressed JEG-3 cells. As seen in Figure 3 and Figure 4, after cells were cultured for 24 h, the mean cell migration distances of the C1/HSPC117, C1, and control cells were 9.46 ± 1.12, 12.89 ± 2.99, and 14.21 ± 0.87 μm, respectively; thus, the *HSPC117* over-expressed JEG-3 migration distance was about 66.57% of that of the control group (*p* < 0.05) and 69.82% of that of the C1 group (*p* < 0.05). Similarly, from 24 to 48 h, the *HSPC117* over-expressed JEG-3 migration distance was about 80.44% of that of the control group (*p* < 0.05) and 83.15% of that of the C1 group (*p* < 0.05). From 48 to 72 h, *HSPC117* over-expressed JEG-3 migration distance was about 74.59% of that of the control group (*p* < 0.05) and 83.15% of that of the C1 group (*p* < 0.05). This may suggest that the migration speed of the control group cells was the fastest, that the C1 cells were slower than the control group cells (*p* > 0.05), and that the C1/HSPC117 cells were slower than both the control group cells and C1 cells (*p* < 0.05). Thus, over-expression of *HSPC117* may cause a decrease in the migration speed of JEG-3 cells.

### 2.2. Discussion

#### 2.2.1. Relationship of *HSPC117* with Epigenetic Inheritance

HSPC117 is an RNA ligase that catalyzes the GTP-dependent ligation of RNA with 5'-OH and either 2',3'-cyclic phosphate or 3'-phosphate ends [15,16]. Both *in vitro* and in living cells, *HSPC117* depletion mediated by RNA interference inhibited maturation of intron-containing pre-tRNA [17,18]. The sequence of the HSPC117 protein family is highly conserved in all domains of life; this suggests that it has RNA ligase roles in various organisms.

When Ygberg *et al.* mutationally inactivated cold-shock-associated exoribonuclease polynucleotide phosphorylase (PNPase) in *S. enterica*, one outcome was an increase in *RtcB* gene expression [19]. *RtcB* depletion by RNAi was correlated with Parkinson symptoms and neurodegeneration in the Caenorhabditis elegans Parkinson disease model. RtcB overproduction resulted in neuroprotection [20]. When mouse *HSPC117* RNAi knock-down embryos were transferred into pseudopregnant females, a large number of embryo deaths were observed after nine days of pregnancy [8]. Therefore, we speculate that *HSPC117* might be influenced by epigenetic modification. However, there are very few reports about its expression regulation by epigenetic modification. To determine if regulation of *HSPC117* mRNA expression was affected by epigenetic modification *in vitro*, JEG-3 cell lines were treated with inhibitors of methylation alone, histone deacetylation alone, or in combination, to examine how those inhibitors influence *HSPC117* expression.

We found it intriguing that the mRNA and protein expressions of *HSPC117* were induced by TSA and 5-aza-dC. The highest expression level was observed when both inhibitors were added to the culture medium; this suggests that *HSPC117* mRNA and protein expressions may be synergistically up-regulated by TSA and 5-aza-dC. Although both inhibitors may regulate *HSPC117* expression, there are still some limitations in our study. Mainly, we did not perform 5-aza-dC and TSA-response elements analysis in the *HSPC117* promoter regulatory region to determine the possible genetic regulation mode of *HSPC117*.

RtcB is the bacterial and archaeal homolog of HSPC117 and RtcA is coregulated by sigma54 in an operon. The region upstream of the transcription start site of the *rtcA/rtcB* mRNA contains -12 TTGCA and -24 TGGCA elements, respectively, which is the characteristic of sigma 54-dependent promoters [2]. Further, other research groups have shown that the human *HSPC117* gene is located on chromosomes 22, and that human Chrs 22 is a CpG-rich island. The CpG islands are important genomic landmarks. They are concentrated in highly acetylated gene rich regions. Many gene expressions are regulated by CpG island epigenetic status. The *HSPC117* promoter also has a CpG island involving the first exon [21]. We suspect that *HSPC117* expression level was up-regulated when cells were treated with 5-aza-dC because the 5-aza-dC decreased the methylation level of CpG islands in the promoter region. Previous studies in leukemia cells have shown several gene expressions can be induced by 5-aza-dC, although their promoters are not directly affected by methylation [22]; this suggests that 5-aza-dC might exert its influence on regulating gene expression through a methylation independent or non-dependent manner. When JEG-3 cells were treated with the HDAC inhibitor, TSA, the expression level of *HSPC117* was slightly increased, while the combination of 5-aza-dC and TSA resulted in a significantly higher expression of *HSPC117* than 5-aza-dC or TSA alone. No report about TSA response element was found in exons of *HSPC117* to its promoter; this indicates that an indirect mechanism might be responsible for TSA induction.

TSA and 5-aza-dC have been widely used for studying epigenetic modification of many genes because of their specific inhibition of histone deacetylation and methylation, respectively [23,24]. By remodeling chromatin via directly converting methylated DNA to unmethylated DNA or unacetylated histones to the acetylated state, TSA or 5-aza-dC usually cause global changes in gene expression, allowing easy access of the transcription machinery to gene promoters [25]. This is why many transcriptional activities of non-histone transcription factors could be affected by the HDAC inhibitor [26].

#### 2.2.2. The Relationship among HSPC117, MMPs, TIMPs, and Cell Migration

There are some lines of evidence that HSPC117 is involved in cell adhesion. However, there is a lack of data regarding the effect of *HSPC117* expression on cell migration. To our knowledge, MMPs and TIMPs participate in the regulation of cellular migration processes by interacting with components of ECM [27]. MMPs degrade components of ECM, while they can be specifically inhibited by TIMPs; thus, MMPs and TIMPs play an important role in remodeling the basement membrane (BM) and ECM [28]. In this process, gelatinases (MMP 2) play an important role, and they have the unique ability to degrade BM. TIMP 2 is a known inhibitor of MMP 2. MMP 14 is known as a membrane-type MMP, which is specifically involved in the processes of cell migration and invasion in a paracrine manner to affect close surroundings [29]. In order to verify that HSPC117 contributes to cell migration through regulation of MMPs and TIMPs, we studied *MMP 2*, *MMP 14*, and *TIMP 2* mRNA expressions in the JEG-3 cells with over-expressed *HSPC117*.

In our study, over-expression of *HSPC117* significantly decreased the expression of *MMP 2* and *MMP 14* mRNA, and expression of *TIMP 2* mRNA apparently increased. We have known that MMP 2 activation at the cell surface requires the participation of MMP 14 and TIMP 2. Through the formation of the trimolecular noncovalent complex of TIMP 2/MMP 14/por-MMP 2 (MMP 2 inactive zymogen pro form), MMP 2 is activated and released to the extracellular space, degrades extracellular matrix gelatin/collagen IV and stimulates cell migration. The concentrations of all three molecules are important in this process [30,31]. MMP 14 can activate MMP 2 in a specific manner, and it is believed that an optimal ratio of MMP 14 to TIMP 2 to activate MMP 2 is in the range of 3:1 to 3:2 [32,33]. It is important to note that over-expression of the *HSPC117* gene changed the proportion of MMP 2, MMP 14, and TIMP 2 in our reverse transcription real-time quantitative PCR (RT-qPCR) study. *MMP 2* and *MMP 14* mRNA declined, which meant a decrease in the amount of the TIMP 2/MMP 14/por-MMP 2 compound, and, as a result, inhibition of MMP 2 activity. Contrarily, *TIMP 2* mRNA significantly increased, and an excess of TIMP 2 may have inhibited the activity of MMP 2. TIMP 2 is not only the inhibitor of MMP 2 but also an inhibitor of MMP 9; both MMP 2 and MMP 9 are enzymes that degrade gelatin. Our results indicate that the ability to degrade gelatin in JEG-3 with over-expressed *HSPC117* declines.

We conducted a scratch wound assay to investigate whether over-expression of *HSPC117* resulted in decreased migration speed through the surface covered with gelatin. The result showed that the migration speed of JEG-3 cells with over-expressed *HSPC117* declined significantly compared to controls, and this is in concordance with our qPCR results. This study lends further support to the supposition that HSPC117 inhibits migration of cells on the surface of the extracellular matrix by modulation of balance between MMP 2, MMP 14, and TIMP 2.

## 3. Materials and Methods

### 3.1. Gene Cloning

Human cervical carcinoma cells were used for the cloning. Total RNA was extracted from human cervical carcinoma cells using a Trizol reagent (Invitrogen, Grand Island, NY, USA) according to the manufacturer’s instructions. cDNA samples were amplified by PCR using specific sense and antisense primers. According to the Human *HSPC117* cDNA sequences (GenBank: NM_014306.4), two pairs of primers F and R were designed. F: 5'-AAGCTTATGAGTCGCAGCTATAATGATGAG-3', R: 5'-GGATCCCTATCCTTTGATCACAGCAATTGGTC-3'. The PCR products were subjected to electrophoresis on a 1% agarose gel, and the expected size of the amplified PCR product was 1518 bp.

### 3.2. Construction and Transfection of EGFP-HSPC117 Expression Plasmid

We used F and R primers to amplify the full coding sequence of the human *HSPC117* gene. HindIII and BamHI restriction sites were incorporated at the 5' ends of the forward and reverse primers. After T-A cloning, the PCR fragment was double-digested using HindIII and BamHI. The product was then inserted into the linear pEGFP-C1 vector, which was digested using the same enzymes, to generate the pEGFP-C1-HSPC117 plasmid, named C1/HSPC117, and the control plasmid was named C1. Subsequently, pEGFP-C1-HSPC117 was sequenced to verify the sequence of the inserted fragment.

Prior to the day of transfection, JEG-3 cells were plated into 6-well plates. When the cells had reached approximately 80% confluence, transient transfections were performed using 10 μL Lipofectamine 2000 reagent (Invitrogen, Carlsbad, CA, USA) with 7 µg of C1 or C1/HSPC117 plasmid DNA. The transfection medium was replaced with normal growth medium after 6 h. HSPC117 mRNA expression in the cells was assessed by RT-PCR. All constructs were confirmed by sequencing.

### 3.3. Cell Culture

JEG-3 cells were cultured in high glucose Dulbecco’s Modiﬁed Eagle’s Medium (DMEM) (Life Technologies Inc., Rockville, MD, USA), supplemented with 10% fetal calf serum in an incubator at 37 °C with 5% (*v*/*v*) CO_2_ and 95% humidity. When the cells reached approximately 80% confluence, they were treated with the following: (1) 5-aza-dC (50 nM) for 3 days; (2) TSA (75 nM) for 1 day; (3) 5-aza-dC (50 nM) for 2 days followed by 5-aza-dC (50 nM) and TSA (75 nM) for 1 day; and (4) complete growth medium without 5-aza-dC or TSA as a control.

### 3.4. RNA Extraction and Quality Control

Total RNA isolated and obtained from each cell line was extracted with TRIzol reagent (Invitrogen, Grand Island, NY, USA), following the instructions of the manufacturer. The concentration and purity of the total RNA were spectrometrically assessed with spectrophotometer Evolution™ 201 (Thermo Scientific Evolution 201, Chicago, IL, USA). The absorbance was measured at 260 and 280 nm. All the RNAs used in this study have the absorbance ratio of A260 nm/A280 nm was between 1.8–2.0, which indicates that the RNA is pure. The concentration of RNA was calculated as follows: RNA concentration (µg/mL) = (OD 260) × (dilution factor) × (40 µg RNA/mL)/(1 OD 260 unit).

In addition, the integrity of the total RNA was assessed by visualization of the 28S/18S band pattern. At least 200 ng of total RNA was loaded onto a 1% denaturing agarose gel stained with ethidium bromide (EtBr) and visualized using GelDoc2000 (Bio-Rad, Hercules, CA, USA). When sharp and clear 28S and 18S rRNA bands were observed, and the 28S/18S rRNA bands have ratio around 2, the total RNA was considered completely intact. The total RNA with confirmed integrity was then used for subsequent experiments.

### 3.5. Reverse Transcription Real-Time Quantitative PCR (PT-qPCR)

One microgram total RNA per sample was used for reverse transcription. The first-strand cDNA was synthesized using M-MLV First Strand Kit (Invitrigon, Grand Island, NY, USA) according to the manufacturer’s protocol, with oligo-dT as primer.

Following reverse transcription, the qPCR amplification was then carried out with the Stratagene (Palo Alto, CA, USA) Mx3000P qPCR system, using the specific primers listed in Table 1 for each gene. Each qPCR reaction was done in a 20 μL volume and contained 10 μL of Power SYBR Green Master Mix (Applied Biosystems, Foster, CA, USA), 0.8 μL of primers (10 pmol), 0.8 μL of template cDNA (50 ng/μL), and 7.6 μL of dd H_2_O. The Stratagene (Palo Alto, CA, USA) Mx3000P cycling conditions were the following: single cycle of 5 min at 95 °C followed by 40 cycles of 30 s at 95 °C, 1 min at 60 °C, and 30 s at 55 °C. PCR amplifications were performed at least three independent biological replicates for each sample to check the reproducibility of the data.

**Table 1:** Gene-specific quantitative PCR (qPCR) primers.

Gene	qPCR Primers	
*HSPC117*	F:	5' ATGACCCTGAAGCAGTAGTATCCC 3'
	R:	5' TACTCCTCGCAGTGCTCCTTGTC 3'
*MMP 2*	F:	5' GTGGATGATGCCTTTGCTCG 3'
	R:	5' CCATCGGCGTTCCCATACTT 3'
*MMP 14*	F:	5' CCCAACATCTGTGACGGGAACT 3'
	R:	5' GAGCAGCATCAATCTTGTCGGTAG 3'
*TIMP 2*	F:	5' CCAAAGCGGTCAGTGAGAAGG 3'
	R:	5' TGGGTGGTGCTCAGGGTGTC 3'
*GAPDH*	F:	5' ACGGATTTGGTCGTATTGGG 3'
	R:	5' CGCTCCTGGAAGATGGTGAT 3'
*β-actin*	F:	5' TCCCTGGAGAAGAGCTACGA 3'
	R:	5' AGCACTGTGTTGGCGTACAG 3'

To determine the stability of the reference genes under experimental conditions, *GAPDH* and *β-actin* mRNA levels in JEG3 cells were measured under control or treated by TSA or 5-aza-dC or *HSPC117* over-expression. The results showed that *GAPDH* and *β-actin* mRNA were not regulated by our experimental conditions, which indicates that *GAPDH* and *β-actin* are perfect reference genes for qPCR in our case. The relative mRNA expression levels of *HSPC117*, *MMP 2*, *MMP 14*, and *TIMP 2* was normalized by *GAPDH* and *β-actin*. The relative qPCR amplification efficiency were 104% (*GAPDH*), 99% (*β-actin*), 97% (*HSPC117*), 103% (*MMP 2*), 99% (*MMP 14*), and 98% (*TIMP 2*), determined by standard curve analysis. It is indicated that 100% can be roughly used as the amplification efficiency, and the relative expression levels were calculated by the formula 2^−ΔΔ*C*t^ where ΔΔ*C*_t_ = (*C*_t_ Target − *C*_t_ *GAPDH/β-actin*) treated − (*C*_t_ Target − *C*_t_ *GAPDH/β-actin*) control.

### 3.6. Western Blot Analysis

JEG-3 cells were washed in phosphate-buffered saline (PBS) and incubated for 30 min on ice in lysis buffer and the lysates were centrifuged at a speed of 16,000× *g* at 4 °C for 10 min. The supernatants were used to measure the protein content using the bicinchoninic acid (BCA) method, and the pellets were heated at 99 °C for 10 min in sodium dodecyl sulfate (SDS) loading buffer. Thirty micrograms of cell lysate protein was electrophoresed by 12% sodium dodecyl sulfate polyacrylamide gel electrophoresis (SDS-PAGE) and then the protein blots were electroblotted to polyvinylidene difluoride (PVDF) membranes.

The membranes with protein blots were blocked with 5% nonfat dry milk for 1 h in Tris buffered saline-Tween (TBST) and then were incubated with primary antibody (1:500 dilution for HSPC117 and 1:2000 for GAPDH/β-actin) overnight at 4 °C. Membranes were incubated at 37 °C with peroxidase-conjugated goat anti-rabbit secondary antibody (1:3000 dilutions) for 1 h at room temperature after being washed in TBST. The blots were detected using the chemiluminescence detection and analysis system.

### 3.7. Scratch Wound Assay

To assay cell migration, JEG-3 cells were transfected with C1/HSPC117 plasmids, C1 plasmids or nothing (control), and seeded in 24-well plates that were coated with diluted gelatin (Sigma, Santa Clara, CA, USA). Once the cells reached confluence, the cells were wounded with a plastic tip that was dragged across the cell monolayer. Cells were incubated in DMEM medium with 1% serum, and the phase contrast images were taken at 0, 24, 48, and 72 h of incubation. Five fields were randomly selected and the distances of migrated cells were measured under a light microscope (Carl Zeiss, Oberkochen, Germany). Experiments were repeated at least three times.

### 3.8. Statistical Analysis

All experiments were performed at least three times, with reproducible results. Statistical analysis was performed using SPSS 15.0 software (SPSS, Chicago, IL, USA). Statistical comparisons were performed with one-way ANOVA. The Tukey multiple comparisons were taken as a *post hoc* test when differences were significant. Differences were considered to be statistically significant when *p* < 0.05.

## 4. Conclusions

Our results indicate that *HSPC117* mRNA and protein expression are regulated by epigenetic modification; inhibitors of methylation (5-aza-dC) and histone deacetylation (TSA) induce the high expression of *HSPC117* mRNA and protein. Further studies indicate that over-expression of *HSPC117* gene reduce *MMP 2* and *MMP 14* gene expression, while *TIMP 2* gene expression was up-regulated, and as the result shows, the speed of JEG-3 cell migration is slower than control group.
